# Later-Life Exposure to Moderate PM_2.5_ Air Pollution and Life Loss of Older Adults in Taiwan

**DOI:** 10.3390/ijerph17061873

**Published:** 2020-03-13

**Authors:** Jing-Shiang Hwang, Tsuey-Hwa Hu

**Affiliations:** Institute of Statistical Science, Academia Sinica, Taipei 11529, Taiwan; jshwang6@stat.sinica.edu.tw

**Keywords:** air pollution health effects, survival extrapolation, long-term PM_2.5_, expected years of life lost

## Abstract

Background: Few studies have directly estimated expected life loss attributable to lifetime exposure to fine particulate matter (PM_2.5_). Methods: We used claims data from Taiwan’s National Health Insurance to create 63 study cohorts of 1.91 million residents aged 60–79 years old residing in small areas where air quality monitoring stations are situated. The survival status of each person was followed from 2001 to 2016. We applied an extrapolation algorithm to estimate the lifetime survival function so that we could directly estimate life expectancy (LE) and the lifetime exposure to PM_2.5_ of each cohort. We estimated the association between LE and lifetime exposure to PM_2.5_ among the 63 cohorts. We also fit a Cox proportional hazards model to all the data combined to estimate the relative risk of mortality. Results: Older adults who lived in an area with a higher lifetime weighted average PM_2.5_ of 10 $\mu g/m^{3}$ had a shortened LE by 0.34 (95% CI: 0.22–0.46) years. The hazard ratio of mortality was 1.0245 (1.0242–1.0248) per one $\mu g/m^{3}$ increase in lifetime average PM_2.5_. Conclusion: This study provides strong evidence that later-life exposure to moderate PM_2.5_ air pollution had a substantial impact on the life loss of older adults.

## 1. Introduction

To improve health risk assessments, the effects on mortality from long-term exposure to ambient particulate matter (PM) air pollution have been well studied [1,2,3,4,5,6,7,8,9,10,11,12,13]. Recently, Pope III et al. complied a comprehensive list of cohort studies in the past 25 or more years and provided substantial evidence of associations between air pollution exposure and mortality through meta-analysis [14]. We have found that most of the cohort studies reported relative mortality risks for an increased unit of PM exposure utilizing Cox proportional hazards models [14]. To have a more meaningful indicator of the health effect of air pollution, the estimated relative risks were used with some assumptions, and under various counterfactual scenarios, to estimate the number of premature deaths and further estimate the loss of life expectancy (LE) attributable to air pollution of a study population [15,16,17].

The expected loss of LE was also used as a primary outcome for quantifying the health effects of long-term exposure to PM_2.5_ in several causal studies [18,19,20,21]. The difference-in-differences approach, a statistical technique to mimic an experimental design by studying the differential effect of exposures in two time periods, was used to evaluate the changes in LE associated with differential changes in PM_2.5_ air pollution between two time periods across US county units [18,19]. Recently, a regression discontinuity design based on the distance between a location and the Huai River in China was proposed to model the relationship between LE and air pollution concentration among selected study areas [20,21].

These studies claimed to provide evidence of the effect on LE from the long-term exposure to PM_2.5_. However, the LE was indirectly calculated by first modeling annual mortality data and population data for each study area, and then using standard life table techniques with the estimated relative risks [19]. The uncertainties of the estimated LE and the effect size of air pollution on LE decrement in the exposed population were rarely discussed. Therefore, more studies with advanced approaches for estimating associations between long-term PM_2.5_ exposure and LE decrement are deeply needed. Furthermore, although these studies examined the health effects of long-term exposure, none of these studies have explored the health impacts of lifetime exposure to PM_2.5_ [14]. This is mainly because few cohorts had their exposure levels and mortality statuses tracked for a sufficiently long period. Without proper approaches for dealing with the high censoring rate problems, it is difficult to obtain robust estimates of LE and lifetime exposure to PM_2.5_ in the study cohorts.

In this study, we proposed a new approach for directly estimating both LE and lifetime exposure to PM_2.5_ in study cohorts of older adults for assessing the health effects of long-term exposure on expected years of life lost. We used Taiwan’s National Health Insurance claims data to create study cohorts of 1.91 million older adults aged 60–79 years who lived or worked in 63 small areas in rural townships and city districts where ambient air quality monitoring stations were situated. We extracted each individuals’ 1998–2000 medical visit records in the claims database to select participants for a study cohort. We then obtained the survival status of the participants from the mortality registry. The participants were followed from the first day of 2001 to the last day of 2016.

We reported an increased risk of mortality attributable to lifetime exposure to PM_2.5_ in two models of mortality at area level and individual level, separately. First, we estimated the lifetime survival function for each study cohort using an extrapolation algorithm [22]. The expected lifetime exposure to PM_2.5_ of each cohort was estimated by adding up the product of PM_2.5_ concentrations in the area that the cohort resided in and survival rates over the lifetime and divided these by the LE of the cohort. We used a weighted regression model with spatially correlated error terms to estimate the effect size on LE by lifetime exposure to PM_2.5_, with adjustments for socioeconomic and demographic variables among the study cohorts in the 63 small areas. Second, we estimated the relative mortality risk of lifetime exposure to PM_2.5_ by fitting a Cox proportional hazards model to the survival data of all the 1.91 million individuals, together with an adjustment for individual risk factors and area-level variables for comparisons with other studies.

## 2. Methods

In addition to analyzing the relative mortality risk of exposure at the individual level using a traditional Cox model, the main focus of the study is the proposed new method for estimating the association between life expectancies and lifetime exposure among study cohorts. The procedures of the new method consist of three steps, including calculating LE and estimating lifetime exposure, both using the lifetime survival function for each cohort, and a linear regression model. We will introduce the extrapolation algorithm for estimating the lifetime survival function before the details of the modeling association between LE and lifetime exposure.

### 2.1. Data Sources

Taiwan’s National Health Insurance was launched in 1995, providing universal health care coverage to 99.6% of Taiwan’s residents and had service contracts with 93% of the country’s hospitals and clinics in 2018 [23]. Data in the registry of beneficiaries comprise a unique encrypted identifier, sex, date of birth and an insured payroll-related amount. The claims data contain diagnoses, prescriptions, and details of inpatient care or outpatient visits. Disease diagnoses are coded using the International Classification of Diseases, Ninth Revision (ICD9). Although the medical facilities have been deidentified, their scale and location can be obtained from the registry.

The Taiwan air quality monitoring network was established in 1993 and collects data from 77 monitoring stations currently. Most monitoring stations are located in populous urban and rural areas and can be used to inform the public on ambient air quality. Open data on hourly measurements of major pollutants are available on the monitoring network website [24]. Data for the township and city district socioeconomic and demographic variables of this study are also available from the Taiwanese government’s open data platform [25]. We also used population and housing census data that were collected in December 2000 to determine the characteristic variables of the selected study areas [26].

### 2.2. Study Design

We selected 63 small study areas, with a median size of 42 km^2^, consisting of rural townships and city districts in Taiwan where air quality monitoring stations are situated and complete ambient PM_2.5_ measurements have been available since 2006. Because ambient monitoring stations are usually located in the populous center of the selected study areas, the PM_2.5_ concentrations measured at the monitoring stations are presumed to represent the air pollution exposure levels of participants living or working in these small areas [27].

For each study area, we created a study cohort of participants who had (1) lived or worked in the area since the start date of January 1, 2001 and (2) had been in the area until the end of 2016 or died during the follow-up. To create a study area cohort, we first identified participants who (1) visited a primary care clinic in the study area for minor treatment during 1998–2000, (2) were living at the end of 2000 and (3) were aged between 60 and 79 years at the start date of January 1, 2001. We defined minor treatment as treatment with a medical fee that was less than the 75th percentile of fees for primary clinic visits. The aforementioned criteria for selecting participants into a study cohort were chosen to exclude participants who resided far away from the study area and who came to a study area for major treatment. Data on all medical visits for each participant during the follow-up period were retrieved. We assumed that a participant in a study area had been living or working in the area from the start to end date; the end date was defined as 12 months after the date of a participant’s last visit to any health facility located within 20 km of the area’s center. If the end date of a participant in an area was later than 2016, it was truncated to the end of 2016. The encrypted identifier for each participant was used to obtain their corresponding survival status data from the mortality registry during the follow-up. A participant’s survival time was defined as the period from the start date to the date of death if the participant visited any health facility located within 20 km of the area’s center within 12 months before death; otherwise, the survival time was defined up until the end date of the participant in the area and the survival time was treated as censored.

### 2.3. Survival Extrapolation Method

Given the survival data of a cohort, the survival function of the cohort, denoted as S(t), can be estimated for some time *t* until the maximum follow-up time. Although the study followed participants aged 60–79 years for 16 years, approximately 50% of the participants were still alive by the end of the follow-up. We required a lifetime extrapolation of the survival function to estimate the LE of the cohort, which is defined as:

(1) $$LE=\int_{0}^{\infty }S\left(t\right)dt.$$

When the censoring rate is high, commonly used parametric models may produce an inaccurate long-term extrapolation of the survival curve of the cohort [28,29]. In this study, we estimated the lifetime survival function using a new method called the rolling extrapolation algorithm [22], which has been demonstrated to be more robust and accurate than popular parametric models when estimating the lifetime survival function of a cohort of patients with specific conditions [30,31].

The use of the rolling extrapolation algorithm in the estimation of LE is detailed in Hwang et al. [22]. Briefly, we described the algorithm in four steps in the following. First, the survival times of referents with sex *s*, age *a* and start year *y* matched with the study participants of the cohort are generated from the survival function:

(2) $$S_{G}\left(t|s,a,y\right)=S_{G}\left(t-1|s,a,y\right)\times \left(1-q_{a+t-1,s}^{y+t-1}\right)\;\mathrm{for}\;t\geq 1,$$

where $q_{a+t-1,s}^{y+t-1}$ is the probability that someone with sex *s* and aged exactly a+t−1 years old will die before reaching age a+t based on the standard life table of year y+t−1 and $S_{G}\left(0|s,a,y\right)=1$. We used these generated survival times for the general referents to obtain the survival function of the general reference population and denoted it as $S_{g}\left(t\right)$. If $S_{g}\left(t\right)$ was larger than S(t), we termed the generated general reference population a healthy reference population and set the survival function of the healthy reference population to be $S_{h}\left(t\right)\equiv \;S_{g}\left(t\right)$. Conversely, if the generated function $S_{g}\left(t\right)$ was not larger than S(t), a proposed method of generating survival times for heathy referents, as detailed in the following, can be used to obtain $S_{h}\left(t\right)$. Let $t_{obs}^{}$ be the observed survival time of a participant with sex *s*, age *a* and year *y* in the cohort. A random number r uniformly distributed between 0 and $S_{G}\left(t_{obs}^{}|s,a,y\right)$ was generated. We then further drew a random number v from Unif(0, r) to obtain a survival time $t_{ref}=S_{G}^{-1}(v|s,a,y)$ for the healthy referent matched with the participant.

Second, we defined a relative survival function between the study cohort and the healthy reference population, denoted as:

(3) $$W\left(t\right)=S\left(t\right)/S_{h}\left(t\right),$$

whose values lie between 0 and 1. Under the assumption of the cohort having an excessive constant hazard of mortality, logit[W(t)] is approximately linear after a certain follow-up time [22,32]. The assumption was reasonable in practice, as demonstrated in the applications [30,31].

Third, the best fitted restricted cubic splines model to the logit[W(t)] curve during the observed period was used to extrapolate the curve one time point ahead. We treated the newly predicted value of logit[W(t)] at the neighboring time point as “observed” because such short-term predictions, with the property of approximate linearity, are usually highly accurate. We then rolled the extrapolation procedures by updating same-length observation periods one time point ahead and refitting the restricted cubic splines models for the updated observation periods to predict the value of logit[W(t)] at the successive time point.

Fourth, when logit[W(t)] was completely extrapolated, we could back-transform it to obtain the complete W(t) function, which was used in conjunction with $S_{h}\left(t\right)$ to calculate an estimate of the study cohort’s lifetime survival function S(t). The survival extrapolation procedures can be implemented using the R package iSQoL2 [33].

### 2.4. Statistical Analysis for Risk at Area Level

The estimated life expectancy of each cohort can be obtained by integrating the extrapolated survival function. The extrapolated survival function can also be used to estimate lifetime exposure of each cohort. We can then fit a linear regression model to the life expectancies and the lifetime exposures among the 63 cohorts to examine the association between exposure and the health outcome. The details of the analysis are described in the following three sub-sections.

#### 2.4.1. Standardized Life Expectancy Deviation

Instead of using LE as the response variable in the linear regression model, we proposed a measure called the standardized life expectancy deviation (SLED) of a study cohort, which is defined as the LE difference between the cohort and the age- and sex-matched general reference population. That is,

(4) $$SLED=\int_{0}^{\infty }S\left(t\right)dt-\int_{0}^{\infty }S_{g}\left(t\right)dt.$$

Similar to the standardized mortality ratio used for comparison among subnational areas in a country, the proposed SLED quantifying the increase or decrease in LE of a study cohort with respect to the general population is better than LE for use in exploring factors determining expected life years gained or lost among the study areas in a country. The estimates and 95% confidence intervals (CI) of LE and SLED can be obtained from the R package iSQoL2 [33].

#### 2.4.2. Expected Lifetime Exposure

The expected lifetime cumulative exposure of a cohort from the start date can be written as $\int_{0}^{\infty }S\left(t\right)\times C\left(t\right)dt$, where S(t) is the extrapolated survival function of the cohort and C(t) is the function of the average concentration of PM_2.5_ air pollution in the study area [34,35]. The expected lifetime exposure to PM_2.5_ of the cohort can then be estimated by the lifetime weighted average, which is denoted by:

(5) $$E=\sum_{t=0}^{T}S\left(t\right)\times C\left(t\right)/LE=\sum_{t=0}^{T}P\left(t\right)\times C\left(t\right),$$

where *T* is the maximum lifetime of the cohort. The weight P(t)=S(t)/LE can be interpreted as the person-times at time *t* divided by the lifetime cumulative person-times. For each time point during a lifespan, the expected lifetime exposure gives weight to PM_2.5_ levels according to the population that is still alive. This weight ensures that the expected lifetime exposure is an appropriate measure of the lifetime exposure to air pollution for a cohort in an area.

#### 2.4.3. Models for the Cohort Measures

As a preliminary measure, let $Y_{i}$ and $s_{i}$ be the estimate of the SLED and standard error (SE) of the estimate of the *i*th study cohort, and let $E_{i}$ be the lifetime weighted average PM_2.5_ for the cohort. We first conducted standard stepwise regression analysis using the model:

(6) $$Y_{i}=\beta_{0}+\beta_{1}E_{i}+\sum_{j=1}^{p}\gamma_{j}X_{ji}+\varepsilon_{i},$$

where $\varepsilon_{i}\;\sim \;N\left(0,s_{i}^{2}\sigma^{2}\right)$. This weighted regression was used to identify the influential covariates $X_{ji}$ from the socioeconomic and characteristic variables. Having identified these covariates, we then re-estimated the coefficients of the selected covariates in the regression model, assuming that the correlation between $\varepsilon_{i}$ and $\varepsilon_{j}$ is $\exp\left(-d_{ij}/\delta \right)$, where the parameter δ denotes the range and $d_{ij}$ is the distance between the centers of the two areas. The R packages stat and rms were used for the analysis.

The covariates included in the stepwise regression model were of three categories, which were grouped according to how their data were obtained. In the first category, data were obtained from the created cohorts, and the covariates were cohort size, mean age at start date, sex proportion, insured payroll-related amount and proportion of the study cohort hospitalized during 1998–2000. In the second category, data were obtained from December 2000 population census data for individuals aged 60 to 79 years, and the covariates were the proportions of college graduates, individuals living with a partner, individuals living with a disability and indigenous people in a study area. In the third category, data were obtained from government-provided open data, and the covariates were population density, proportion of adults older than 60 years during 1996–2000, gross consolidated income, number of hospital beds, and number of regional hospitals and medical centers located within 20 km of the area’s center.

### 2.5. Statistical Analysis for Risk at Individual Level

We fit a Cox proportional hazards model to all the data of the 1.91 million participants in the 63 cohorts to estimate the hazard ratio of the lifetime average PM_2.5_ of these older adults. The lifetime exposure of a participant is given by the average of monthly mean PM_2.5_ concentrations of the area the participant lived or worked in during the period from 12 months before the start date of January 1, 2001, to the month the participant died or their data were censored. The Cox model was adjusted for age, sex, insured payroll-related amount, any hospitalization due to diabetes, hypertension, cardiovascular diseases, hypercholesterolemia, chronic obstructive pulmonary disease and other diseases of the individuals during the 3 years before the start date, and area-level variables listed above.

## 3. Results

The characteristic variables of the 63 study areas and corresponding cohorts are summarized in Table 1. The sizes of the study areas had a median of 42.4 km^2^ and a range of 1.9–247.2 km^2^. The population densities of the study areas ranged widely from 26,562 people per km^2^ for a district in Taipei City to 200 people per km^2^ for a rural township; the median was 2,149 people/km^2^. The largest study cohort had 136,512 people, whereas the smallest cohort had 1,993 people; the median cohort size was 23,323 people. Average ages at the start date of follow-up ranged between 67.4 and 68.7 years. The proportions of women were as low as 44% and as high as 76%.

**Figure 1:**
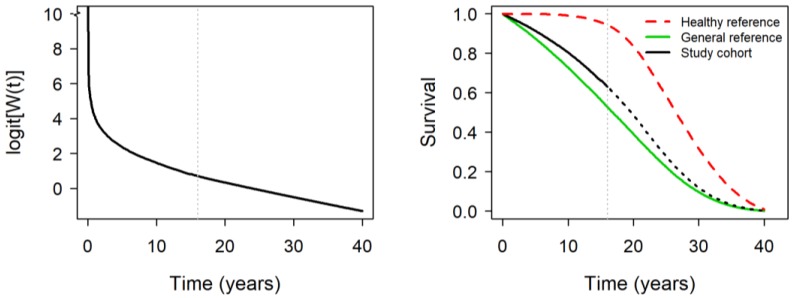
Extrapolated logit[W(t)] curve and survival functions for a study cohort and matched reference populations.

To obtain insight into the workings of the proposed survival extrapolation method, the extrapolated logit[W(t)] curve and extrapolated survival curve for the study cohort of Sonsang district in Taipei City are plotted in Figure 1. The cohort had superior survival than its matched general reference population before the maximum follow-up of 16 years. We, therefore, generated another survival curve for the healthy reference to define the relative survival function W(t) for extrapolating the survival curve of the cohort. The logit[W(t)] curve was approximately linear before the end of the maximum follow-up and beyond. With the extrapolated survival curve, we obtained an estimate of 19.0 (SE = 0.10) years for LE and 1.98 (0.10) years for SLED. The median LEs of the 63 study cohorts of participants aged 60–79 years was 17.1 years and their LEs ranged between 15.6 and 19.3 years. The SLEDs of the cohorts ranged between -1.3 and 2.0 years.

The hourly PM_2.5_ and PM_10_ concentrations during 2006–2018 are available for the 63 study areas from the air quality monitoring network; PM_10_ data for the years before 2006 are also available. We aggregated the measurements to obtain monthly average concentrations of PM_2.5_ for 2006–2018 and PM_10_ for 2001–2018 for each study area. The annual average concentrations of PM_2.5_ ranged between 14.8 and 50.7 μg/m^3^ and had a median of 32.0 μg/m^3^ among the 63 study areas in 2006; while the annual concentrations of PM_2.5_ gradually reduced to a range of 6.8–27.7 μg/m^3^ and a median of 19.7 μg/m^3^ in 2018. The ranges of annual concentrations of PM_10_ were 33.6–90.4 μg/m^3^ and 25.9–65.9 μg/m^3^ among the 63 study areas in 2006 and 2018, respectively. The correlation coefficient between the monthly concentrations between PM_2.5_ and PM_10_ during 2006–2018 was 0.9.

We used a fitted linear model of measured monthly levels of PM_2.5_ that were regressed against PM_10_ concentrations for 2006–2018 to estimate monthly average concentrations of PM_2.5_ from 2001 to 2005 for each study area. We then used a fitted autoregressive integrated moving average time series model to the observed monthly mean PM_2.5_ levels in 2006–2018 to predict the monthly mean levels after 2018 for each study area. Because of the clear downward trend of PM_2.5_ air pollution in Taiwan, the model-predicted monthly mean levels were decreasing for all 63 areas. We set a lower bound of 10 μg/m^3^, the annual average concentration chosen by the World Health Organization as the long-term guideline value for PM_2.5_, for the predicted monthly levels in an area if the area’s annual average concentration in 2018 was larger than 10 μg/m^3^. If the area’s annual average concentration was smaller than 10 μg/m^3^ in 2018, this annual average concentration was the lower bound. The expected lifetime exposure to PM_2.5_ for the 63 study cohorts, which ranged from 12.0 to 38.6 μg/m^3^, had a median of 26.9 μg/m^3^.

**Figure 2:**
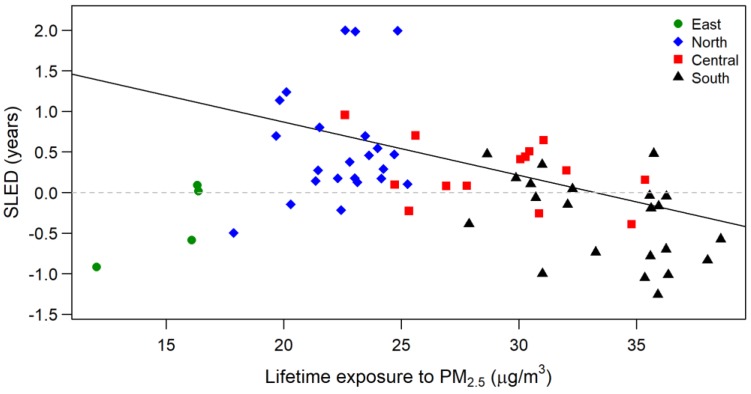
Standardized life expectancy deviation plotted against expected lifetime exposure to particulate matter (PM_2.5_) for the cohorts in the 63 study areas, which are marked with four different shapes to indicate cohorts located in eastern, northern, central and southern Taiwan. The slope of the added solid line is −0.066.

Figure 2 presents the plot of SLED against expected lifetime exposure to PM_2.5_ for the 63 study cohorts. The simple regression model of SLED against expected lifetime exposure yielded a slope of approximately −0.066 (0.015). However, the plot also exhibited clear clustering for study areas in northern, southern, and eastern Taiwan. The association between SLED and expected lifetime exposure level was further estimated, with influential covariates adjusted for using the stepwise regression model.

Table 2 summarizes the estimated coefficients for the association between PM_2.5_ levels and SLED, in addition to the effects of five selected covariates from the final weighted regression model with spatially correlated error terms. When the effects of influential socioeconomic and demographic covariates were controlled for, the coefficient estimate of lifetime exposure was −0.034 (0.006). Although the association estimate in the model of SLED against the single variable of PM_2.5_ exposure was very strong (Figure 2), the adjusted R^2^ value was only 0.22. The stepwise regression model increased the adjusted R^2^ to 0.87, indicating that the estimated effect size of PM_2.5_ exposure was appropriately adjusted by the five selected covariates (Table 2).

**Figure 3:**
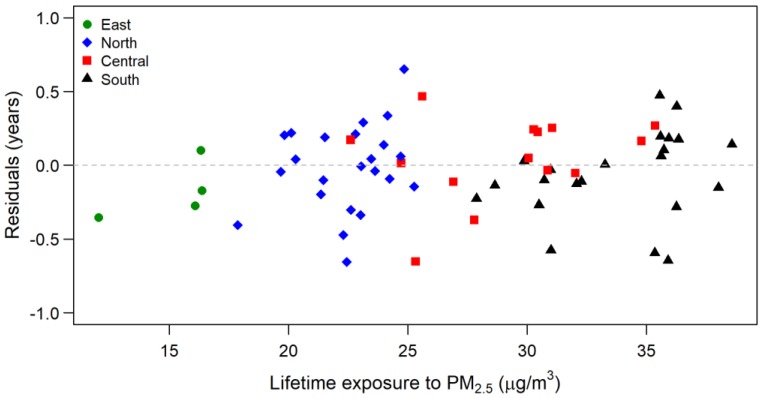
Residuals of the fitted regression model plotted against expected lifetime exposure to PM_2.5_ for the cohorts in the 63 study areas.

It is not surprising that the proportion of a cohort being hospitalized during the 3 years before follow-up was negatively associated with SLED. This indicates that health conditions at the start date affected LE in the follow-up. We also determined that socioeconomic condition—represented by the proportion of elderly people with a college degree in a cohort—was positively associated with the SLED of a cohort (*p*-value < 0.001). According to Table 2, the higher the proportion of people aged 60 years during 1996–2000, the higher the cohort’s LE in the follow-up. This implies that those living in an area with more older residents are likely to live longer. It is also interesting to find that older people who lived with a partner also had a significantly longer LE than those without partners (*p*-value < 0.001). Medical resources, represented by the number of regional hospitals and medical centers within 20 km of an area’s center, were positively associated with the SLED of a cohort. According to the residuals plot of the final model in Figure 3, the location clustering effect was considerably lower than that at the initial stage. This indicates that the confounding effects were largely eliminated by the selected covariates.

As a comparison with related studies, we observed that a 10 $\mu g/m^{3}$ increase in lifetime weighted average PM_2.5_ was significantly associated with an estimated mean LE loss of 0.34 (0.06) years for the adults aged 60–79 years. To estimate the effect of lifetime exposure to PM_2.5_ on expected years of life lost among the study cohorts, the annual average concentration of 10 $\mu g/m^{3}$ was set as a baseline. The largest excessive lifetime exposure of the 63 cohorts from the baseline was 28.6 $\mu g/m^{3}$. The expected years of life lost was 0.97 years for older adults living in the study area that had the worst air quality since 2001. The life loss relative to the cohort’s LE of 15.9 years was 6.1%.

There were 1.91 million people in the 63 cohorts at the start date of January 1, 2001. By the end of 2016, 804,000 (42%) had died. After the adjustment for all the available individual risk factors and area-level variables in the Cox proportional hazards model, the estimate of the hazard ratio of mortality for each one μg/m^3^ increase in lifetime average PM_2.5_, was 1.0245 (95% CI: 1.0242–1.0248). The result was very close to the hazard ratio of 1.021 (1.019–1.022) reported in a similar cohort study of long-term exposure to PM_2.5_ and mortality among older adults in US [36].

## 4. Discussion

The comprehensive claims data of Taiwan’s National Health Insurance and the wide range of PM_2.5_ levels that spanned more than 10 years enabled us to propose this new study design and set of analytical approaches for examining the association between lifetime exposure to PM_2.5_ and LE. Our results showed that the increase in lifetime weighted average PM_2.5_ was significantly associated with an estimated mean LE loss based on modeling the standardized life expectancy deviations against the exposure of the cohort. We also fit the same weighted linear model with the response variable SLED replaced by LE. The estimated association between expected lifetime exposure to PM_2.5_ and LE was very close to the estimate of the SLED modeling after the adjustment for the additional two variables of average age at the start date and proportion of females in the cohorts. Although the conclusions of the two models were the same in this study, we prefer to report the results of the SLED modeling, as the two additional variables of age and sex may be correlated with the other explanatory variables in the model of LE.

Although the study cohorts of people aged 60–79 years were followed for 16 years, their survival rates at the end of 2016 ranged between 46.6% and 63.2% (Table 1). The accuracy of the extrapolation of the survival function to the end of life is critical for estimating LE. The rolling extrapolation algorithm has been demonstrated to be more accurate and robust in terms of long-term survival extrapolation [22]. However, this algorithm assumes that the study cohort has a lower survival than its matched general reference population. In this study, we modified this algorithm to generate a reference population that is always healthier than the matched study cohort for survival extrapolation. To evaluate the performance of this proposed modification, we assumed that the survival status of the 63 study cohorts were followed to the end of 2010. We then extrapolated the survival function to the end of 2016. The difference between the observed and extrapolated survival functions to the end of 2016 yielded a survival time difference that ranged from −2.2 to 1.8 months among the 63 cohorts. Therefore, our modified algorithm still has an excellent performance with a mean relative absolute error of 0.43%.

Our analysis of hazard ratio for mortality using the claims data of 1.91 million residents was very similar to that of the study of 13.1 million Medicare beneficiaries residing in seven southeastern US states [36]. Both of the studies fit Cox proportional hazards models with adjustments for available confounding variables. The estimated hazard ratios for death in the two studies were very close, with 1.0245 and 1.021, respectively, per one μg/m^3^ increase in lifetime average or yearly mean PM_2.5_. The difference is that the median exposure level was 26.9 μg/m^3^ in our study, which was moderate relative to key representative studies of the health effects of long-term PM_2.5_ exposure [37], as it was 10.7 μg/m^3^ in the US study. This indicated a linear relationship of mortality risk among the elderly with annual PM_2.5_ in the range of 10–27 μg/m^3^. Our estimate of association between lifetime exposure to PM_2.5_ and LE is also consistent with the findings of a relevant study that modeled differences in LE against differences in PM exposure over time. Our result of a decrease of 10 μg/m^3^ in lifetime average PM_2.5_ was associated with a mean LE increase of 0.34 (0.06) years, which was close to the 0.35 (0.16) years measurement obtained in the analyses of data in 545 US counties for the period from 2000 to 2007, with annual PM_2.5_ down from 13.2 to 11.6 μg/m^3^ [19]. The difference is that our estimated effect size was for assessing expected later-life loss of older adults, while the US study was for examining the change of LE at birth in two time periods.

Although we included several influential socioeconomic variables in our model, our results may be affected by other unmeasured confounders. Thus, we cannot infer causality between lifetime exposure to PM_2.5_ air pollution and LE loss in this study. Furthermore, the strong correlation between concentrations of PM_2.5_ and other pollutants, especially ozone, among the 63 study areas hindered us from fitting data on multiple pollutants in the model. At least for our single-pollutant model, we found that LE was also strongly associated with the annual mean concentration of ozone. It is generally difficult to determine which air pollutants were responsible for LE loss based on our findings. However, the strong evidence obtained from our analysis further strengthens our belief that later-life exposure to air pollution shortens LE among older adults.

## 5. Conclusions

The proposed survival extrapolation algorithm allows for the accurate estimation of LE and lifetime exposure to air pollution of a cohort, even when the censoring rate is high. Using comprehensive data on health, air quality, and socioeconomic status, we presented strong evidence that expected lifetime exposure to PM_2.5_ is associated with the LE of people aged 60–79 years old in Taiwan. Both the proposed methods and findings of our study contribute to health impact assessments of long-term exposure to air pollution.

## Figures and Tables

**Table 1:** Summary characteristics of the 63 study areas and corresponding cohorts.

Variable	Min	25%	50%	75%	Max
*Variable related to the study cohorts*					
Cohort size (people)	1993	13029	23323	44312	136512
Age at start date (year)	67.4	67.7	67.9	68.1	68.7
Female of the cohort (%)	44.1	51.7	54.0	55.8	75.8
Insured payroll-related amount (in NT$1000)	15.4	18.8	19.5	20.1	22.2
Hospitalization during 1998–2000 (%)	11.4	13.4	14.4	16.1	20.1
Survival rate at the end of follow-up	46.6	51.4	53.7	55.5	63.2
*Area variables calculated from census data collected in December 2000*					
Age 60–79 living with a partner (%)	65.5	71.3	72.5	74.4	76.9
Age 60–79 living with severe disability (%)	4.6	5.4	5.9	6.4	8.3
Age 60–79 with a college degree (%)	0.4	1.4	2.8	4.2	24.9
Age 60–79 who is indigenous people (%)	0.0	0.0	0.1	0.3	14.2
*Area variables retrieved from government open data*					
Area size (km^2^)	1.9	29.1	42.4	70.0	247.2
Population density (people/km^2^) ^a^	200	1080	2149	5927	26562
Age 60+ in 1996–2000 (%)	5.3	9.2	11.9	13.4	18.6
Gross consolidated income (in NT$1000) ^b^	605	735	784	879	1672
Number of hospital beds (x 100) ^b^	0	2.78	6.53	14.27	50.38
Number of large hospitals within 20 km ^c^	0	9.5	21	44	67

The data used for analysis come from different periods: ^a^ 2010, ^b^ 2013, ^c^ 2016.

**Table 2:** Effect estimates of influential variables associated with standardized life expectancy deviation (years) from the weighted regression model with spatially correlated error terms.

Variable	Estimate	Std. Error	*p*-Value
Hospitalization of the cohort 1998–2000 (%)	−0.071	0.022	0.002
Number of large hospitals within 20 km	0.009	0.003	0.002
Age 60+ of the area 1996–2000 (%)	0.041	0.015	0.010
Age 60–79 living with a partner (%)	0.126	0.018	0.000
Age 60–79 with a college degree (%)	0.060	0.011	0.000
Lifetime weighted average PM_2.5_ ($\mu g/m^{3}$)	−0.034	0.006	0.000
